# Contrast-Enhanced Ultrasound Guided Biopsy of Undetermined Abdominal Lesions: A Multidisciplinary Decision-Making Approach

**DOI:** 10.1155/2017/8791259

**Published:** 2017-01-04

**Authors:** Feng Mao, Yi Dong, Zhengbiao Ji, Jiaying Cao, Wen-Ping Wang

**Affiliations:** ^1^Department of Ultrasound, Zhongshan Hospital, Fudan University, 200032 Shanghai, China; ^2^Shanghai Institute of Medical Imaging, 200032 Shanghai, China

## Abstract

*Aim.* To investigate the value of contrast-enhanced ultrasound (CEUS) guided biopsy of undetermined abdominal lesions in multidisciplinary treatment (MDT) decision-making approach.* Methods.* Between Jan 2012 and Dec 2015, 60 consecutive patients (male, 37; female, 23; mean age, 51.3 years ± 14.6) who presented with undetermined abdominal lesions were included. CEUS and core needle percutaneous biopsy was performed under real-time CEUS guidance in all lesions. Data were recorded and compared with conventional ultrasound (US) guidance group (*n* = 75). All CEUS findings and clinical data were evaluated in MDT.* Results.* CEUS enabled the delimitation of more (88.3% versus 41.3%) and larger (14.1 ± 10.7 mm versus 32.3 ± 18.5 mm) nonenhanced necrotic areas. More inner (20.0% versus 6.7%) and surrounding (18.3% versus 2.7%) major vessels were visualized and avoided during biopsies. CEUS-guided biopsy increased the diagnostic accuracy from 93.3% to 98.3%, with correct diagnosis in 57 of 60 lesions (95.0%). The therapeutic plan was influenced by CEUS guided biopsies findings in the majority of patients (98.3%).* Conclusion.* The combination of CEUS guided biopsy and MDT decision-making approach is useful in the diagnostic work-up and therapeutic management.

## 1. Introduction

Undetermined abdominal lesions may represent a clinical challenge as a conclusive diagnosis may be difficult, especially in patients with no evidence of primary neoplastic disease [1]. Imaging modalities can provide useful diagnostic information but cannot determine whether a lesion is benign or malignant.

Imaging guided core needle biopsy of suspected abdominal lesions is popular because it can have a major effect on patient management; diagnostic surgical procedures can be avoided, and planning for therapy can be accelerated [2–5]. Diagnostic interventional ultrasound (INVUS) procedures are efficient, minimally invasive techniques. Ultrasound (US) is the ideal imaging modality to guide interventional procedures with several advantages: the absence of radiation and lack of potentially nephrogenic contrast agents; US is inexpensive and real-time imaging ensures the visualization of needles, to guide a needle in real-time into organs, masses, and lymph nodes [6], thus improving diagnostic accuracy with a reduction of complications [2–5]. The current EFSUMB guideline of IVUS procedures recommends that in the case of indeterminate retroperitoneal masses (e.g., sarcoma), the indication for biopsy versus primary resection should be individually assessed (LoE 4, GoR C, and strong consensus 100%) [3]. An ultrasound retroperitoneal core biopsy is more accurate than fine needle aspiration and should be performed whenever possible (LoE 3b, GoR C, and broad agreement 84%) [3]. However, in previous studies, conventional US guided percutaneous biopsy results were accurate in 75–100% of patients. Indeed, the lack of cellular atypia because of paucity of material collected by US guided biopsy is the main reason for nondiagnostic findings and represents a crucial point in the workup of a large abdominal lesion. In such cases, repetition of US guided biopsy may be used to enhance diagnostic accuracy [7].

Contrast-enhanced ultrasound (CEUS) guided percutaneous biopsy is a new developed technique aimed at increasing the accuracy of percutaneous biopsies [8]. The use of CEUS may be helpful in large tumors with necrosis or in tumors that are invisible or poorly visible on grayscale US to improve the accuracy in obtaining adequate tissue samples [8, 9].

Clinically, the multidisciplinary team (MDT) setting should be the standard to discuss INVUS procedures to confirm the necessity of the procedure, possible alternatives, and complications [3]. Interdisciplinary ultrasound is typically shaped by the interaction of surgeons, radiologists, pathologists, and oncologists departments in a dedicated effort to provide more accurate diagnostic and effective therapeutic strategy for patients [10].

The aim of our study was to assess the impact of CEUS guided biopsy in the management of patients with suspected abdominal lesions in a MDT decision-making approach.

## 2. Materials and Methods

### 2.1. Institutional Board Approval

This prospective study was approved by our institutional review board. All patients gave their full informed consents before the CEUS examination and IVUS biopsy. The procedure followed was in accordance with the Declaration of Helsinki.

### 2.2. Patients and Lesions

Between January 2012 and December 2015, 60 consecutive patients (male 37, female 23, mean age, 51.3 years ± 14.6) who presented with undetermined abdominal lesions with interdisciplinary ultrasound treatment during MDT were included in the study (Table 1).

The inclusion criteria were patients referred to the Department of Ultrasound in our institution for ultrasound guided percutaneous biopsy of undetermined abdominal lesions, which were detected on contrast-enhanced MRI or CT within one month. Lesion larger than 25 mm in diameter was accessible via ultrasound guided percutaneous biopsy approach.

Exclusion criteria were a contraindication to the ultrasound contrast agent SonoVue (Bracco Imaging Spa, Milan, Italy) (e.g., severe cardiopulmonary function or known allergic reactions) or a contraindication to core needle aspiration (bleeding tendency, prothrombin activity < 40%, international normalized ratio > 1.7, or platelet count < 40,000/mL).

Seventy-five consecutive patients (male 43, female 32, mean age, 53.8 years ± 15.8) who underwent conventional ultrasound guidance (US group, *n* = 75) were also retrospectively analysed.

### 2.3. Ultrasound Examination Procedures

Conventional US, CEUS, and further ultrasound guided biopsy procedures were performed with HD15 units (Philips, Bothell, WA, USA) and a C5-2 broadband curved transducer. All ultrasound examinations were performed by one physician with 20 years' experience in IVUS on the same day. The clips were recorded, stored, and reviewed by the two readers, who reached a consensus with emphasis on the presence of necrotic and viable areas. The display rates of necrotic areas and viable areas in lesions were recorded and compared between two groups.

Prior to biopsies, US were first performed to detect the lesion, to observe the lesion's echogenicity, size, and location. Patients were lying in supine position with arms lifted above heads. We evaluated the possibility of the core needle biopsy sampling and chose the optimal acoustic window for further CEUS examination (CEUS group) or biopsy route. In conventional US group, color Doppler flow images (CDFI) were performed to identify major vessels in lesions, which should be avoided during further biopsy. The pulse repetition frequency and wall filters were adjusted to enable the better display of intralesional vessels and to avoid “blooming” artefacts. “Necrotic areas” in US group were defined as anechoic area with no color flow signal. “Viable areas” in US group were defined as echoic areas with relatively rich color flow signal. For patients with multiple abdominal lesions, the biggest one which was suitable for further CEUS or biopsy procedure was observed.

Typically, a two-step algorithm was used for the CEUS procedure, a preliminary injection of contrast agent to identify the lesion and plan the intervention strategy, and a second injection to perform CEUS guided biopsy procedure. During each CEUS examination, 2.4 mL SonoVue (Bracco, Milan, Italy) was injected in quick bolus via a 20-gauge intravenous catheter placed in the cubital vein, followed by a flush of 5 mL normal saline 0.9%. The mechanical index (MI) was set to 0.05–0.10. Focus was positioned at the bottom of the screen to minimize microbubble destruction. Field of view and gain were optimized to provide the best depiction of the lesion. The enhancement patterns (homogeneous or inhomogeneous), internal necrotic or viable areas, and major vessels inner or surrounding lesions were recorded. “Viable areas” were defined as the most obviously enhanced or perfused regions inner lesions. “Necrotic areas” were anechoic regions without enhancement.

### 2.4. US or CEUS Guided Core Needle Biopsy Procedure

Ultrasound guided core needle biopsies of abdominal lesions were performed on the same day after the optimal biopsy routes and sampling sites were selected, targeting at the viable area and avoiding the necrotic area or major vessels. After skin was sterilized, the predicted needle path was anesthetized with 2% lidocaine, and core needle biopsies were performed with real-time ultrasound guidance. We used 16-gauge core tissue biopsy needle and Bard® Magnum® biopsy instrument (Bard Peripheral Vascular Inc., USA). Patients were instructed to suspend respiration when the needle was advanced into the target area. In CEUS group, during the arterial phase of CEUS, the needle was advanced into the enhanced viable areas, while avoiding the large unenhanced necrotic areas or major vessels.

Two 15 mm long core specimens were obtained for each patient. The biopsy specimen was placed on a small piece of filter paper. The operator checked and evaluated whether the specimen was adequate. After being immersed in 10% formalin, the specimens were sent for histological examination.

Conventional US were performed to search for any possible complication such as localized hematoma or pneumothorax after core needle biopsy procedure. Patients were closely monitored for 2 to 4 hours.

### 2.5. Histological Examination

All histopathologic slides were reviewed by one 15-year experienced pathologist. Hematoxylin and eosin (HE) staining and microscopic observations were performed for the histopathologic diagnosis. Immunohistochemical and electron microscopy studies were performed when necessary.

### 2.6. Multidisciplinary Team Approach for Clinical Evaluation and Decision-Making Strategy

CEUS guided core needle biopsy findings of abdominal lesions, together with all other clinical data, were evaluated in a weekly MDT meeting together with gastroenterologists, surgeons, oncologists, radiologists, and pathologists. Based on all valid clinical data, MDT defined the surgical or nonsurgical strategy (chemotherapy, clinical follow-up) for each patient. Chemotherapy was started based on a diagnosis of advanced neoplastic disease or lymphoma. Patients' data, including operation, further investigations, or clinical follow-up were recorded in individual files.

For those patients with nondiagnostic CEUS guided biopsy findings (e.g., lack of enough cellular features suggesting malignancy), surgery was considered if the suspicion of malignancy was highly established by clinical data.

### 2.7. Final Diagnoses

During a 6-month clinical follow-up, the final diagnoses were established. For the true-positive CEUS guided biopsy findings, malignancies were confirmed by surgery or rebiopsy specimens, and benign diagnoses were established on chest CT scans during follow-up, which showed shrink or disappearance of the abdominal lesion after certain treatment. False-negative diagnoses were considered in the event of nondiagnostic CEUS guided biopsy: that is, CEUS guided biopsy findings were negative for malignancy, while subsequent surgery or rebiopsy specimens confirmed malignancy without definite diagnosis of benign lesions (diagnosis such as chronic inflammation and necrosis), or biopsies without adequate specimens. The diagnostic accuracy of CEUS guided biopsy was defined as the percentage of lesions that had true-positive histopathological results by initial biopsy.

### 2.8. Statistical Analysis

Data were expressed as mean ± standard deviation. The difference in the diagnostic accuracy between two groups was analysed by using Student's *t*-test. A difference was considered statistically significant with *P* < 0.05. All statistical analyses were performed with SPSS 17.0 software package (SPSS, Chicago, IL, USA).

## 3. Results

### 3.1. Comparison of Ultrasound Features between US and CEUS Groups

In US group, CDFI detected branched inner lesion vessels in 57.3% (43/75) lesions; the mean value of RI in Doppler spectrum was 0.65 ± 0.41. Inner necrotic areas as anechoic area in different size were defined in 41.3% (31/75) patients (Table 2).

**Figure 1:**
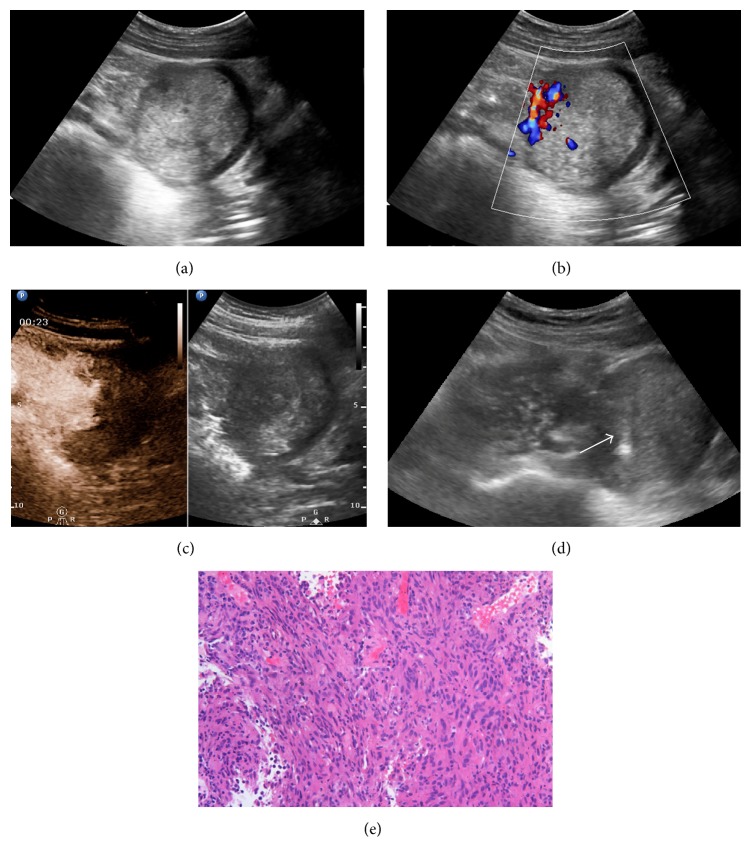
Grayscale ultrasound detected a heterogeneous hypoechoic abdominal lesion in a 48-year-old male. No necrotic area was shown with conventional ultrasound (a). CDFI detected blood flow in the peripheral area of the lesion (b). In CEUS, large and irregular nonenhanced necrotic area was displayed inner lesion (c). With CEUS guidance, 16-gauge coarse needle percutaneous biopsy (arrow) was performed successfully in the viable areas of the lesion (d). Histopathological results proved it was a gastrointestinal stromal tumor (e).

**Figure 2:**
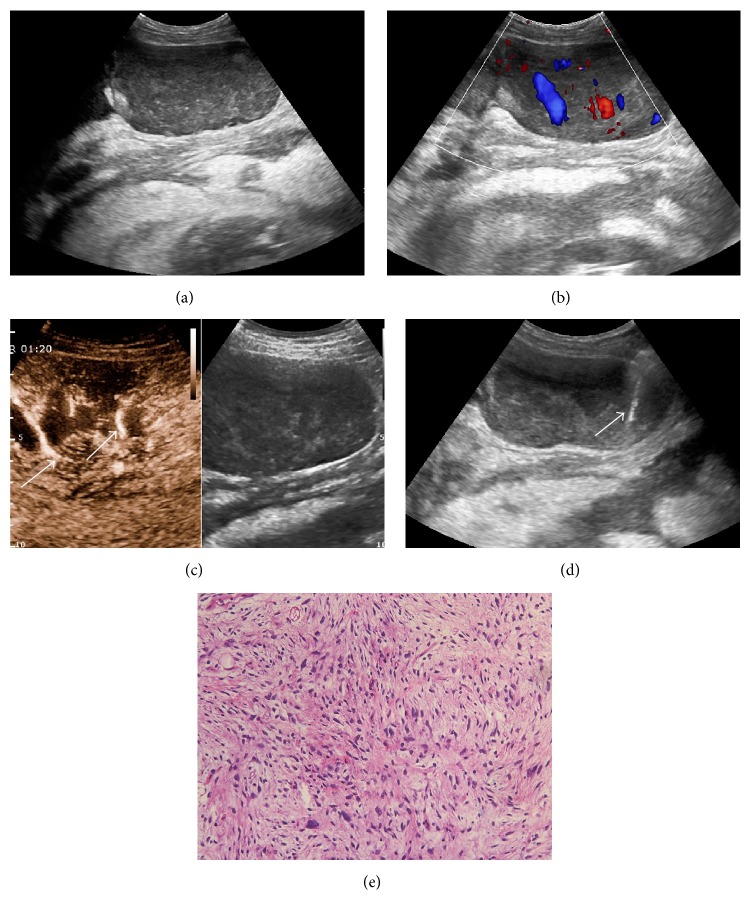
Grayscale ultrasound detected a heterogeneous hypoechoic abdominal lesion in a 43-year-old female. No necrotic area was shown with conventional ultrasound (a). CDFI detected multiple blood flow inner lesion (b). In CEUS, major vessel was displayed inner lesion (arrow) (c). With CEUS guidance, 16-gauge coarse needle percutaneous biopsy (arrow) was performed successfully in the viable areas of the lesion and avoided major vessel (d). Histopathological results proved it was a liposarcoma (e).

In CEUS group, CEUS examinations were successfully performed in all 60 patients; no adverse reaction of SonoVue was observed. After injection of SonoVue, all 60 abdominal lesions showed inhomogeneous hyperenhancement. Unenhanced inner necrotic areas were clearly displayed in 88.3% (53/60) lesions, which was higher than US group (88.3% versus 41.3%, *P* < 0.05) (Figure 1). During the early arterial phase of CEUS enhancement (5–10 sec after injection of contrast agents), special attention was paid to the rapid and obviously hyperenhanced major artery inner or surrounding the abdominal lesions in 38.3% (23/60) patients, which were effectively avoided during further biopsy (Figure 2).

Good interreader agreement (*κ* = 0.793) was achieved after two observers reviewed and discussed the CEUS clips of all cases.

### 3.2. Comparison of Biopsy Success Rate between US and CEUS Groups

In CEUS group, we advanced needles during arterial phase of CEUS by targeting the obviously hyperenhanced viable areas. In abdominal lesions showing obvious unenhanced necrotic areas on CEUS, biopsies were performed avoiding the necrosis. Meanwhile, we arranged the safe biopsy routes by avoiding major large vessels. In 1 case with large necrotic area, the core needle biopsy specimen was not adequate for histopathological analysis.

In US group, conventional ultrasound successfully guided percutaneous biopsies with 16-gauge core needles in 70 abdominal lesions. In 5 cases, the core needle biopsy specimen was inadequate for histopathological analysis.

There was a significant difference between the CEUS group and the US group in the rate of successful puncture attempts (Table 2). None of the patients had adverse reactions or biopsy complications.

### 3.3. Comparison of Diagnostic Power between US and CEUS Guided Biopsy

When compared with final surgical histopathology or clinical follow-up data, the initial CEUS guided biopsy led to correct diagnosis in 57 of 60 lesions (95.0%). None of the 57 diagnoses established on the CEUS guided biopsy findings were false positive, whereas 3 were found to be false-negative results, including one hematoma (inadequate specimen), one seminoma (initial biopsy diagnosed as mesothelioma), and one liposarcoma (initial biopsy diagnosed as aggressive fibromatosis). Thus, the diagnostic accuracy of CEUS guided biopsy was 95% (Table 3).

In US group, the initial biopsy led to correct diagnosis in 66 of 75 lesions (88.0%). False-negative diagnoses were obtained in 3 patients because of necrosis of the biopsy specimen; in 5 patients because of inadequate specimen; and in one patient because of no definite benign diagnosis (schwannoma). The difference in the diagnostic accuracy between the CEUS and US groups was statistically significant (*P* < 0.05).

### 3.4. Multidisciplinary Team Approach of the Decision-Making Strategy and Clinical Impact of CEUS Guided Biopsy

Clinical impacts of CEUS guided biopsy on the MDT decision-making strategy of abdominal lesions are shown in Table 4.

CEUS guided biopsy results achieved correct diagnoses in 57 patients (95%). It allowed changing the initial clinical diagnoses from malignant to benign in 9 patients (15.8%) and confirmed malignancy in 47 patients (82.4%). Among 47 patients with malignancy in CEUS group, MDT correctly decided to change the early clinical surgical plan in 15 patients based on CEUS guided biopsy results, as surgery was not required or not appropriate for lymphoma (*n* = 14) or metastasis malignancies (*n* = 1). Early indication to surgery was confirmed in 40 patients (31 patients with malignant diagnoses and 9 patients with benign diagnoses). CEUS guided biopsies results provided the opportunity to define targeted chemotherapy in 16 patients based on their immunohistochemical results.

In the remaining 4 patients with benign or nondiagnostic findings, clinical follow-up was conducted for at least 6 months; regular clinical examination or CT scan was used to assess disease progression.

## 4. Discussion

Undetermined abdominal lesions cause symptoms or become palpable only when they have reached a significant large size [11]. The histological results of core needle biopsies are essential for accurate and precision therapeutic and diagnostic purposes in MDT decision-making approach [1]. In a considerable proportion of patients (25–29%) with uncertain origin of the lesion, the management of the disease did not require surgery [11]. Histological verification of the origin of the lesion is an essential prerequisite for further management [2]. In patients suffering from advanced neoplasia or lymphoma, detailed histological information of the tumor is necessary to offer evidence supporting the MDT decision of a proper treatment, such as chemotherapy, new molecular, or gene oncological therapies [8].

Up to now, percutaneous US guided biopsy is a minimally invasive approach often required for large abdominal tumors, when cross-sectional imaging results are nondiagnostic in clinical practice [4–6]. Its major advantages over CT guidance include being flexible choice of puncture route, allowing real-time visualization of the biopsy needle and its tip, being less expensive, and not using ionizing radiation as in CT-guided interventions [7, 12].

However, when the abdominal lesions have huge dimensions with heterogeneous areas on conventional ultrasound, it is difficult to identify the most representative viable area(s) for sampling. Previously, the overall diagnostic rate of US guided core needle biopsy was 88.5% for retroperitoneal tumors [13]. For sarcomas and lymphomas, the sensitivities are lower: 82% and 87%, respectively [14, 15]. The nondiagnostic rate is most often related to insufficient material for diagnosis, which may cause unnecessary delay in diagnosis and treatment, repeated biopsies, or increased costs [2]. Some authors suggested that the performance of percutaneous biopsy in abdominal tumors may be improved using larger needles (especially for lymphoma subtyping) [16], more passes [8, 16, 17], and avoiding necrotic areas [8].

A CEUS guided intervention can be performed in much the same way as routine US guided procedure [13, 18]. CEUS has been shown to be helpful in tumor localization during ultrasound guided biopsy [8, 15, 19] and increase the accuracy of percutaneous needle biopsy in tumor diagnosis by targeting hyperperfused viable areas inner lesion [8, 9]. For instance, previously literature showed that biopsy sampling success rate and pathological diagnosis rate were 100% and 98.1% with CEUS guided percutaneous biopsy in peripheral pulmonary lesions [9].

In our current study, CEUS can reliably evaluate the potentially viable areas before biopsy, by depicting the viable obvious enhancement area after injection of contrast agents. Meanwhile, CEUS is also effective in real-time guiding the biopsy needle to sample a more representative tissue specimen. Comparing to conventional US guided biopsy, CEUS guided biopsy increases the diagnostic accuracy by 5% (98.3% versus 93.3%) and decreases the false-negative rate in those large undetermined abdominal lesions.

Necrotic tissue cannot be identified on conventional US, especially before liquefaction has occurred, possibly leading to an unsuccessful biopsy [8]. Previously, imaging guided biopsy was always performed in the peripheral zone in larger lesions tumors to avoid false-negative diagnosis [8]. On CEUS, the necrotic areas usually present no enhancement in all vascular phases comparing to the surrounding enhanced parenchyma. According to recently published EFSUMB guidelines on INVUS, CEUS can be helpful to avoid necrotic areas in percutaneous biopsy of intra-abdominal tumors. (LoE 3b, GoR C, and strong consensus (100%)) [4, 5]. In our current study, comparing to the conventional US guidance, CEUS enabled the delimitation of more (88.3% versus 41.3%) and larger (14.1 ± 10.7 mm versus 32.3 ± 18.5 mm) nonenhanced necrotic areas from viable vascularized areas inner lesions during different phases of CEUS. Thus, CEUS was used as real-time guidance to avoid necrotic areas while targeting the needle into those undetermined abdominal lesions to ensure the biopsy success rate.

Although major bleeding complications are very rare, percutaneous US guided interventions are associated with a low bleeding risk of injury to large vessels (with subsequent intraperitoneal bleeding) [12]. With real-time US guidance, core needle biopsy may be performed through a safe needle track and avoiding major vessels in large abdominal tumors [15, 16]. With CEUS, more inner (20.0% versus 6.7%) and surrounding (18.3% versus 2.7%) major vessels of abdominal lesions were visualized and avoided in our series. Also, number of puncture attempts had been significantly decreased while comparing to conventional US guidance (2.75 ± 0.55 versus 2.00 ± 0.05). CEUS precisely guided the needle tip during the whole biopsy procedure and eliminated the potential risk of bleeding complications.

According to the histological results of CEUS guided biopsies and tumor size, MDT evaluated the potential resectability of tumors and the probability of chemotherapy (lymphoma, GIST), in order to establish a correct diagnosis and an appropriate management in the management of patients with a suspected abdominal neoplastic lesion [11, 16, 20]. In our series, the therapeutic plan was influenced by CEUS guided biopsy findings in the majority of patients (98.3%). CEUS guided biopsy findings contributed to avoiding surgery in 26.7% of patients, defining a targeted chemotherapy in 26.7% of patients, and confirming surgical approach in 66.7% of patients. CEUS guided biopsy could confirm or alter the early diagnosis of malignancy diagnosed by CT or MRI in 78.3% of patients. All these data showed that CEUS guided biopsy had a significant impact on the MDT decision-making strategy in the management of patients with undetermined abdominal lesions, which strongly influenced the diagnostic workup and therapeutic management of those patients. More invasive and expensive surgical ways, such as laparoscopy, may be avoided. Furthermore, for those patients with nondiagnostic CEUS guided biopsy, MDT could help to make a correct diagnosis and an appropriate therapeutic management or clinical follow-up.

## 5. Conclusion

In conclusion, this study demonstrates that percutaneous CEUS guided biopsy is an efficient, minimally invasive, accurate, and safe method in the diagnosis of undetermined abdominal lesions. CEUS guided biopsy had a significant impact on the MDT decision-making strategy. MDT evaluation allows overcoming some CEUS guided biopsy methodological limitations. The combination of CEUS guided biopsy and MDT decision-making approach is useful in the diagnostic workup and therapeutic management of those patients.

## Figures and Tables

**Table 1:** Baseline characteristics of patients in two groups.

Characteristic	CEUS group (*n* = 60)	US group (*n* = 75)
Age (year)		
Mean ± SD	51.3 ± 14.6	53.8 ± 15.8
Range	17–83	16–80
Male/female	37/23	43/32
Underlying diseases		
Previous tumor history	7	8
None	53	67
CEA (ng/mL)		
≤5, *n* (%)	45 (75.0%)	34 (45.3%)
>5, *n* (%)	15 (25%)	41 (54.7%)
CA 19-9 (*μ*/mL)		
≤4.9, *n* (%)	25 (41.6%)	51 (68.0%)
>4.9, *n* (%)	35 (58.4%)	24 (32.0%)
Initial clinical diagnose		
Malignant, *n* (%)	56 (93.3%)	70 (93.3%)
Benign, *n* (%)	4 (6.7%)	5 (6.7%)

CEA: carcinoembryonic antigen; CA19-9: carbohydrate antigen 19-9.

**Table 2:** Comparison of ultrasound features and biopsy success rate between US and CEUS groups.

Characteristic	US group (*n* = 75 patients)	CEUS group (*n* = 60 patients)	*P* value
Size (mm)	127.3 ± 54.3	131.8 ± 96.5	0.54
Location			
Intraperitoneal	64 (85.3%)	48 (80.0%)	0.36
Retroperitoneal	11 (14.7%)	12 (20.0%)	0.65
BMUS features			
Hypoechoic	65 (86.7%)	55 (91.7%)	0.21
Solid-cystic	10 (13.3%)	5 (8.3%)	0.31
CDFI features			
Inner lesion vessels	43	37	0.44
RI	0.65 ± 0.41	0.71 ± 0.24	0.09
Inner necrotic area			
Number	31 (41.3%)	53 (88.3%)	0.04
Size (mm)	14.1 ± 10.7	32.3 ± 18.5	0.03
Display of major vessels			
Inner lesion major vessels	5 (6.7%)	12 (20.0%)	0.03
Surrounding major vessels	2 (2.7%)	11 (18.3%)	0.02
Number of puncture attempts	2.75 ± 0.55	2.00 ± 0.05	0.04
Biopsy success rate	93.3% (70/75)	98.3% (59/60)	0.04

BMUS: B mode ultrasound; CDFI: color Doppler flow imaging; US: ultrasound; RI: resistance index; CEUS: contrast-enhanced ultrasound.

**Table 3:** Histological diagnoses of biopsies in US and CEUS groups.

Characteristic	US group (*n* = 75 lesions)	CEUS group (*n* = 60 lesions)
Malignant	58 (77.3%)	47 (78.3%)
Lymphoma	31	15
Malignant gastrointestinal stromal tumor	4	5
Liposarcoma	5	9
Synovial sarcoma	2	3
Ganglioneuroma	2	1
Seminoma	2	1
Mucinous adenocarcinoma	2	2
Leiomyosarcoma	2	3
Neuroendocrine carcinoma	3	4
Malignant mesothelioma	1	2
Metastatic adenocarcinoma	2	1
Renal clear cell carcinoma	2	1
Benign	12 (16.0%)	12 (20.0%)
Gastrointestinal stromal tumor	6	4
Solitary fibrous tumor	3	2
Schwannoma	1	3
Chronic inflammation	2	2
Hematoma	/	1
Inadequate specimen	5 (6.7%)	1 (1.7%)

US: conventional ultrasound; CEUS: contrast-enhanced ultrasound.

**Table 4:** Impact of CEUS guided biopsy on the MDT decision-making strategy in CEUS group.

Clinical MDT decisions based on CEUS guided biopsy findings	CEUS group (*n* = 60)
Exclusion of malignancy	9 (15.0%)
Confirmation of malignancy	47 (78.3%)
Avoiding surgery	16 (26.7%)
Confirming surgery	40 (66.7%)
Targeted Chemotherapy	16 (26.7%)
Clinical follow-up	4 (6.7%)

CEUS: contrast-enhanced ultrasound; MDT: multidisciplinary team.
